# Serological Evidence of Phleboviruses in Domestic Animals on the Pre-Apennine Hills (Northern Italy)

**DOI:** 10.3390/v13081577

**Published:** 2021-08-10

**Authors:** Davide Lelli, Vittorio Scanferla, Ana Moreno, Enrica Sozzi, Valentina Ravaioli, Maria Renzi, Giovanni Tosi, Michele Dottori, Antonio Lavazza, Mattia Calzolari

**Affiliations:** Istituto Zooprofilattico Sperimentale della Lombardia e dell’Emilia Romagna, Via Bianchi 9, 25124 Brescia, Italy; vittorio.scanferla@izsler.it (V.S.); anamaria.morenomartin@izsler.it (A.M.); enrica.sozzi@izsler.it (E.S.); valentina.ravaioli@izsler.it (V.R.); maria.renzi@izsler.it (M.R.); giovanni.tosi@izsler.it (G.T.); michele.dottori@izsler.it (M.D.); antonio.lavazza@izsler.it (A.L.); mattia.calzolari@izsler.it (M.C.)

**Keywords:** phlebovirus, sand flies, arbovirus, serology, virus neutralization, Toscana virus, Fermo virus

## Abstract

Phleboviruses are arboviruses transmitted by sand flies, mosquitoes and ticks. Some sand fly-borne phleboviruses cause illnesses in humans, such as the summer fevers caused by the Sicilian and Naples viruses or meningitis caused by the Toscana virus. Indeed, traces of several phleboviral infections have been serologically detected in domestic animals, but their potential pathogenic role in vertebrates other than humans is still unclear, as is the role of vertebrates as potential reservoirs of these viruses. In this study, we report the results of a serological survey performed on domestic animals sampled in Northern Italy, against four phleboviruses isolated from sand flies in the same area. The sera of 23 dogs, 165 sheep and 23 goats were tested with a virus neutralization assay for Toscana virus, Fermo virus, Ponticelli I virus and Ponticelli III virus. Neutralizing antibodies against one or more phleboviruses were detected in four out of 23 dogs, 31 out of 165 sheep and 12 out of 23 goats. This study shows preliminary evidence for the distribution pattern of phleboviral infections in different animal species, highlighting the potential infection of the Toscana virus in dogs and the Fermo virus in goats.

## 1. Introduction

Many phleboviruses (*Phenuiviridae* family) are arboviruses transmitted by sand flies. The genus *Phlebovirus* currently includes more than 60 species [1]. Several of these cause illnesses in humans, such as the febrile illness caused by *Sicilian phlebovirus* (SFSV) and *Naples phlebovirus* (SFNV) [2] (also known as three-day fevers), or *Toscana phlebovirus* (TOSV)-induced meningitis [3,4]. Cases of TOSV infection have been recorded in numerous Mediterranean countries, underlining an extensive areal diffusion from Spain to Turkey [3]. 

Many phleboviruses have been detected in domestic animals, although their pathogenic role remains unclear [5] or has only been recognized years after their effective discovery; indeed, the first evidence that TOSV caused disease in humans was reported 12 years after its initial isolation [6]. The epidemiology of phleboviruses is enigmatic; for instance, the existence of vertebrate reservoirs is still debated. In recent years, several new phleboviruses have been discovered; however, for the majority of these, their pathogenic potential in both humans and other animals is still unknown [4].

Several phleboviruses have been isolated and detected in sand flies in Emilia-Romagna and Lombardy, especially in areas known to be suitable for these insects (such as the pre-Apennine hills) [7]. Among these viruses, we count the well-known TOSV, first isolated from *Phlebotomus perniciosus* in central Italy in 1971 [2] and then detected in Emilia-Romagna in more recent years [8]. The Fermo virus (FERV) was isolated for the first time, in 2012, from *Phlebotomus perfiliewi*, collected in the Fermo area (Marche region) [9]. Moreover, the first isolation of Ponticelli viruses I, II and III (PONV-I, PONV-II and PONV-III) occurred, in 2013, in Emilia-Romagna [10]; these three closely related viruses differed in their M segment, probably as a result of re-assortment. While the PONVs belong to the species *Adana phleboviruses*, FERV represents a species on its own [11,12]. Therefore, its pathogenic potential is unknown, as is its environmental cycle and possible vertebrate reservoirs. In an attempt to clarify their viral ecology, we used these isolated viruses to detect specific neutralizing antibodies (ntAbs) in serum samples collected from domestic animals using a virus neutralization assay (VNT), for the routine diagnostic activities of the Istituto Zooprofilattico Sperimentale della Lombardia e dell’Emilia Romagna (IZSLER), in the same areas in which the above-mentioned viruses were isolated in recent years. 

## 2. Materials and Methods

### 2.1. Sampling

**Figure 1:**
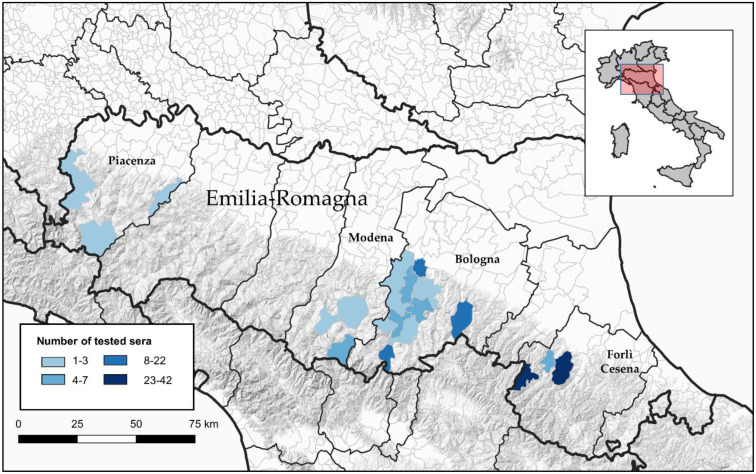
Map of municipalities of sample collection sites of tested animals.

A total of 211 serum samples from different animal species (dogs, goats and sheep), collected in 2019, were tested by a VNT for the viral strains of interest. We obtained these samples by exploiting IZSLER diagnostic activities (especially for the control of sheep brucellosis), mainly in the pre-Apennine hills, where viruses used in VNTs have been isolated (Figure 1). Specifically, the 211 serum samples from dogs (23), sheep (165) and goats (23) originated from the provinces of Bologna (18 dogs, 37 sheep and 23 goats), Forlì-Cesena (5 dogs and 109 sheep), Modena (9 sheep) and Piacenza (10 sheep).

### 2.2. Virus Neutralization Assay (VNT)

The serum samples were tested by a microtiter VNT performed with four phleboviruses including the (i) the TOSV strain VR181135-14/2013 (GenBank KU573064-KU573066), (ii) the FERV strain 212236/2018, (iii) the PONV-I strain VR181135-4/2013 (GenBank KX388223-KX388225), and (iv) the PONV-III strain VR220116-5/2013 (GenBank KX388208-KX388210). All these viral strains were isolated from pools of sand flies [10,13]. Briefly, 25 µL of each serum, with a serial two-fold dilution series of 1/5 to 1/40, was added to two wells of a flat-bottom tissue culture microtiter plate and then incubated for 1 h at 37 °C with an equal volume of tissue culture fluid containing 100 TCID_50_ of each virus. A virus back titration of the working dilution of the virus was included, using six wells per ten-fold dilution, to confirm the validity of the test results. After one hour, a volume of 50 µL of Vero cells (cell culture biobank of IZSLER, code BSCL 6) at a log_(10)_ 4 cells/mL concentration was added to each well and incubated at 37 °C with 5% CO_2_ for 96–120 h. The wells were observed under an inverted microscope to evaluate the degree of the cytopathic effect (CPE) compared with the virus control. The neutralizing titers were expressed as the reciprocals of the final serum dilutions required to neutralize more than 90% of the CPE in the inoculated cultures. A sample was considered to be positive when the CPE was reduced by more than 90% at the lowest dilution (1/10) of serum.

### 2.3. Statistical Analysis

Statistical analysis was performed using MedCalc^®^ Statistical Software version 13.1.0 (MedCalc Software Bvba, Ostend, Belgium; http://www.medcalc.org accessed on 23 July 2019).

The Chi-square test was performed in order to obtain information on the significance of the observed differences between the number of positive and negative VNT results for each of the viral agents among the different animal species sampled in the study.

## 3. Results

Among the 211 serum samples collected from dogs (23), sheep (165) and goats (23), 47 serum samples (4 dogs, 12 goats and 31 sheep) showed neutralizing activity for one or more phlebovirus strains (TOSV, FERV, PONV-I and PONV-III) in the VNT (Table 1). 

In more detail, among the 23 analyzed dog serum samples, four samples (17.3%) (all coming from the Bologna province) showed the presence of ntAbs against TOSV and, possibly, other phleboviruses. Specifically, sample 233414 showed reactivity for TOSV and FERV, with the same ntAb titers of 20; sample 167629/3 tested positive, with an ntAb titer of 20 for TOSV and a lower titer (i.e., 10) for both PONV-I and PONV-III; sample 167629/2 tested positive for TOSV and PONV-I, with the same ntAb titer of 20; and sample 162679/3 only reacted against TOSV (Ab titer, 10) (Table 1).

Among the 21 goat serum samples, 12 samples (57.1%) (collected in Bologna province) tested positive for one or more phlebovirus strains. The highest reactivity in terms of the number of positive samples (11/12) and highest ntAb titer (≥80) was obtained against FERV. There were only two samples that showed the highest ntAb titers for PONV-III, while one sample neutralized TOSV, PONV-I and PONV-III at a 1/20 dilution but was negative for FERV (Table 1). 

Among the 165 analyzed sheep serum samples, 31 samples (18.7%), collected in four different provinces (Bologna, Forlì-Cesena, Modena and Piacenza), showed neutralizing activities for at least one of the four phlebovirus strains included in the study. In detail, 18 samples tested positive for FERV, with ntAb titers ranging from 10 to ≥80. Thirteen samples reacted against TOSV, with Ab titers between 10 and 40. Seven samples showed reactivity against PONV-I and PONV-III, with Ab titers ranging from 10 to 40. Among the sheep serum samples, 20 samples showed neutralizing activity against only one virus strain, while 11 samples neutralized multiple phleboviruses (Table 1).

The significance of the observed differences between the number of positive and negative VNT results for each of the viral agents among the different animal species sampled in the study is reported in Table 2.

## 4. Discussion

TOSV ntAbs have been detected in domestic animals across the Mediterranean basin in areas suitable for sand flies. The results of this study on testing animal serum samples are consistent with the findings of other studies in other Mediterranean countries, such as Portugal [14], Algeria [15], Turkey [16], Corsica [17] and Spain, where TOSV infections have also been detected in horses, cats, sheep, pigs and cattle [18], as well as in Kosovo, where TOSV infections were also detected in cattle and sheep [19]. Whether domestic ani-mals can act as natural reservoirs of infection or can play a key role in TOSV ecology is still unclear. According to a recent study, healthy domestic dogs are not highly susceptible to infection by TOSV or SFSV and do not develop significant viremia or excrete the virus following infection. Consequently, dogs are unlikely natural reservoir hosts of infection and do not appear to play a substantial role in phleboviral transmission cycles [5]. However, dogs can be considered to be potential sentinels for exposure to TOSV transmission by sand flies in the Mediterranean region.

We performed a preliminary serosurvey in domestic animals against three phleboviruses other than TOSV. The high prevalence of ntAbs against FERV detected in goats (47.8%, 11/23) as compared with those obtained in sheep (10.9%, 18/165) and dogs (4.3%, 1/23) was of particular interest. This allows us to speculate that goats could potentially act as a reservoir for this virus, or that this animal is particularly susceptible to the FERV; however, we do not have an indication of the potential pathologies caused by this virus. 

This study showed the VNT reactivity of serum samples from different animals against TOSV, FERV, PONV-I and PONV-III. The epidemiological interpretation of this result is difficult due to (i) the limited information available for these recently discovered viruses; (ii) the low titers against both viruses obtained in many cases; and (iii) the possibility of serological cross-reactivity.

It is noteworthy that the VNT is considered to be the most stringent and specific serological assay currently available for testing serum samples for phleboviruses. However, we cannot exclude the fact that the presence of ntAbs at titers as low as 10 may have been caused by cross-reactivity with other phleboviruses possessing similar antigenic patterns to the ones tested [17,18,19].

In the absence of an officially recognized and standardized cut-off value for the neutralization assays for phleboviruses, we analyzed serum samples starting from the dilution 1/10, describing the sample reactivity for each of the viruses, without definitively attributing a positive result. However, other authors established higher cut-off values for what might be considered a positive neutralization result [20,21].

Despite the presence of TOSV ntAbs in several animal serum samples, the role of non-human vertebrates as reservoirs or amplification hosts remains unclear, since the vertical transmission of phleboviruses in sand flies could be sufficient to maintain the virus in the environment [22,23]. In addition, the recently discovered routes of horizontal transmission may play a relevant role in the circulation of phleboviruses in nature [24].

The evidence of phlebovirus circulation among domestic animals observed in this study should be considered for future investigations in order to extend the current knowledge on the viral ecology and potential pathogenicity of these viruses in domestic animals. Phlebovirus infections can be asymptomatic, but the likely symptoms, similar to those caused by other phleboviruses (e.g., Rift Valley fever virus) in animals [25], range from depression, anorexia, fevers, hemorrhagic fevers and neurological symptoms, to abortions and malformed fetuses. Clues for a possible pathogeny for humans with PONV-II were obtained from patients in Lombardy, who showed neurological symptoms ranging from mild (disorientation and confusion) to severe (meningitis and meningoencephalitis) [26]. 

It is worthwhile mentioning that, to date, a vertebrate reservoir for the sand fly-borne phleboviruses included in this study has not been identified; thus, serological surveillance for FERV and other phleboviruses in animals is epidemiologically useful as an indirect indication of exposure to the virus and of the intensity of circulation in a particular region. 

In conclusion, this study indicates the diffusion of four different phleboviruses in domestic animals, highlighting a possible role for goats as a reservoir or preferential amplification host for FERV. This preliminary evidence represents a starting point for planning broader serological surveys and experimental studies to confirm this hypothesis and to characterize the potential pathogeny of this virus and the other tested viruses. 

## Figures and Tables

**Table 1:** Neutralizing antibody titers against four phlebovirus strains.

					NT Antibody Titers	
Species	Id	Municipality (Province)	TOSV	FERV	PONV-I	PONV-III
Dog	167629/2	Zola Predosa (BO)	10	<10	10	<10
Dog	167629/3	ValsaMoggia (BO)	20	<10	10	10
Dog	162679/3	Monte S. Pietro (BO)	10	<10	<10	<10
Dog	233414	Nr (BO)	20	20	<10	<10
Goat	166082/1	MarzaBotto (BO)	20	20	10	10
Goat	166082/2	MarzaBotto (BO)	20	≥80	10	<10
Goat	166082/3	MarzaBotto (BO)	10	≥80	10	40
Goat	166082/4	MarzaBotto (BO)	20	40	10	80
Goat	166082/5	MarzaBotto (BO)	10	<10	10	10
Goat	173004/1	Castel di Casio (BO)	10	≥80	<10	<10
Goat	173004/2	Castel di Casio (BO)	<10	40	<10	<10
Goat	172838/1	Zola Predosa (BO)	<10	10	<10	20
Goat	172838/2	Zola Predosa (BO)	10	40	<10	<10
Goat	172838/3	Zola Predosa (BO)	10	≥80	<10	<10
Goat	172838/4	Zola Predosa (BO)	40	≥80	20	20
Goat	172838/5	Zola Predosa (BO)	20	40	<10	20
Sheep	164918/1	Fanano (MO)	<10	<10	<10	20
Sheep	164914/2	Lama Mocogno (MO)	<10	20	<10	10
Sheep	165509/1	Monterenzio (BO)	20	10	<10	<10
Sheep	165509/2	Monterenzio (BO)	20	20	20	40
Sheep	165509/4	Monterenzio (BO)	<10	20	<10	<10
Sheep	175269/1	Monterenzio (BO)	<10	10	<10	<10
Sheep	175269/2	Monterenzio (BO)	10	<10	<10	<10
Sheep	175269/4	Monterenzio (BO)	10	<10	20	<10
Sheep	175269/6	Monterenzio (BO)	<10	<10	20	<10
Sheep	175269/9	Monterenzio (BO)	40	10	20	<10
Sheep	175269/10	Monterenzio (BO)	<10	10	<10	<10
Sheep	175269/11	Monterenzio (BO)	<10	<10	<10	10
Sheep	175269/12	Monterenzio (BO)	<10	10	20	<10
Sheep	175269/13	Monterenzio (BO)	20	<10	<10	<10
Sheep	175269/14	Monterenzio (BO)	<10	10	<10	<10
Sheep	235798/3	Predappio (FC)	<10	10	<10	<10
Sheep	235798/4	Predappio (FC)	40	20	<10	<10
Sheep	235798/5	Predappio (FC)	40	<10	<10	<10
Sheep	235798/7	Predappio (FC)	40	≥80	20	<10
Sheep	235798/8	Predappio (FC)	<10	10	<10	<10
Sheep	235798/9	Predappio (FC)	<10	20	<10	<10
Sheep	235798/10	Predappio (FC)	40	<10	<10	<10
Sheep	235798/12	Predappio (FC)	10	<10	<10	<10
Sheep	235798/18	Predappio (FC)	10	<10	<10	<10
Sheep	235798/26	Predappio (FC)	<10	10	<10	<10
Sheep	235798/38	Predappio (FC)	<10	<10	<10	10
Sheep	235798/40	Predappio (FC)	20	<10	<10	<10
Sheep	256991/1	Vernasca (PC)	<10	40	20	<10
Sheep	263514/1	Pecorara (PC)	<10	<10	<10	10
Sheep	263517/1	Bobbio (PC)	<10	40	<10	10
Sheep	263517/2	Bobbio (PC)	<10	40	<10	<10

**Table 2:** Prevalence of neutralizing antibodies for each virus and species.

Virus	No. of Sera with ntAbs Divided by Species (% of ntAbs)			Significance Level *p* Value *
	Dog	Sheep	Goat	
TOSV	4/23 (17.3%)	13/165 (7.8%)	10/23 (43.4%)	*p* < 0.0001
FERV	1/23 (4.3%)	18/165 (10.9%)	11/23 (47.8%)	*p* < 0.0001
PONV-I	2/23 (8.6%)	7/165 (4.2%)	6/23 (26%)	*p* = 0.0006
PONV-III	1/23 (4.3%)	7/165 (4.2%)	7/23 (30.4%)	*p* < 0.0001

* *p* values of less than 0.05 indicate statistically significant difference.

## Data Availability

Not applicable.
